# The Low Congruence between Plant and Animal Diversity in Field Ridges of Intensively Managed Paddy Landscapes, China

**DOI:** 10.3390/plants13121680

**Published:** 2024-06-18

**Authors:** Yicheng Peng, Haoyu Qiu, Yuyang Qian, Jiaxin Chen, Guoyu Qin, Pengyao Li, Rongqian Zhang, Meichun Duan

**Affiliations:** 1 College of Agronomy and Biotechnology, Southwest University, Chongqing 400715, China; pycchen9@163.com (Y.P.); qiuhaoyu1102@163.com (H.Q.); qianyuyang0611@163.com (Y.Q.); 16600256722@163.com (J.C.); 2 Engineering Research Center of South Upland Agriculture, Ministry of Education, Chongqing 400715, China; 3 Department of Plant Sciences, Wageningen University & Research, 6700 Wageningen, The Netherlands; guooyuuu@gmail.com; 4 School of Architecture, Southwest Minzu University, Chengdu 610041, China; li.pengyao@swun.edu.cn; 5 College of Resources and Environmental Sciences, Southwest University, Chongqing 400715, China

**Keywords:** vascular plant, spiders, carabids, birds, species composition, intensive management, cross-taxon congruence

## Abstract

Field ridges are commonly viewed as the stable semi-natural habitats for maintaining plant diversity in the agricultural landscape. The high plant diversity could further support higher animal diversity. But following the adoption of well-facilitated farmland construction measures in China, many field ridges have been disproportionately neglected or destroyed. Empirical studies delineating the relationships between plant and animal diversity in these field ridges in the paddy landscape remain scant, especially in China, which has the most rice production. A two-year field ridge evaluation was conducted in the Chengdu Plain area, covering 30 paddy landscapes. This investigation scrutinizes the shape attributes of field ridges, their plant diversity, and the associated animal α-diversity and community compositions, including spiders, carabids, birds, frogs, and rice planthoppers. In the results of Pearson’s correlation analysis, a significant inconsistent correlation was observed between plant diversity and animal diversity. The analysis of community structure heterogeneity also revealed no correspondence for species composition between plant and animal communities (i.e., spiders, carabids, and birds), while the non-metric multidimensional scale analysis indicated a substantial difference in the species composition of spiders or plants even within the same field ridge between 2020 and 2021. We argue that the implementation of intensive management practices in paddy landscapes, such as machine ploughing and harvesting and herbicide spraying with drones, leads to a scarcity of stable animal and plant communities in field ridges. Therefore, besides retaining these field ridges in paddy landscapes, maintaining the long-term stable ridges by refraining from herbicide spraying or artificial weeding, as well as avoiding winter wheat cultivating in field ridges, will contribute to protecting biodiversity of field ridges as semi-natural habitats.

## 1. Introduction

Over the past few decades, as more farmlands have been used intensively to produce agricultural products, synergizing ample production with biodiversity conservation has become a great challenge in agricultural practice [1,2,3,4]. Agriculture intensification often leads to habitat loss and decreased species diversity, which exacerbates the difficulty of achieving these dual goals. Concurrently, modern intensive agriculture characterized by mechanized production results in homogenized farmland landscapes, further contributing to biodiversity loss. On the other hand, the biodiversity is crucial for maintaining ecological balance, enhancing ecosystem health, and driving the sustainable development of agriculture worldwide [5,6,7]. The non-crop semi-natural habitats within farmlands show promise in mitigating biodiversity loss, countering the detrimental effects of intensive production [6,8,9]. Therefore, these habitats are vital for maintaining the stability and multi-functionality of farmland ecosystems [8,9].

Field ridges, which are common boundaries between two adjacent fields, can serve as semi-natural habitats in paddy landscapes [10]. They are commonly covered by rich vegetation and could provide abundant refuges, overwintering and breeding habitats, and survival resources for the animals of farmlands. Much research across the world has verified the important role of the field ridges for biodiversity conservation in agricultural landscapes [11,12,13,14]. Nevertheless, the bulk of pertinent research is centered around dryland agricultural landscapes, such as those of wheat, soybeans, and corn [15,16]. The field ridges of paddy lands are not only integral for retaining water and serving as pivotal linear dividers between different owners’ paddy fields but also for providing habitats for many organisms, thereby protecting farmland biodiversity [17,18]. The unique aquatic environment of paddy fields endows the field ridges with the potential to serve as exclusive habitats for a diverse range of hygrophilous plants, arthropods, and amphibians [19]. Exploring the biodiversity and ecological importance of paddy field ridges is crucial for understanding their role in agricultural ecosystems [17,20].

The animals’ diversity commonly links to plants in ecosystems. Habitats with higher plant diversity provide more food types and quantity, as well as diverse microhabitat options for different animals, helping to attract and sustain a wider range of animal communities. Higher plant diversity in habitats further supports complex food webs, increasing animal diversity [21,22,23], also called cross-taxon congruence [24] or button-up control. This interplay between plant and animal diversity helps maintain and strengthen the stability of the entire ecosystem [25]. However, there are few studies on whether the paddy field ridge can maintain a stable relationship between animals and plants. Furthermore, would the attributes and structural characteristics of the ridges affect the correlation between plants and animals? 

Spiders, carabids, birds, and frogs play vital roles in agricultural ecosystems. Spiders and carabids can control pest populations [26,27]. Birds contribute similarly by eating a wide range of agricultural pests [28]. Frogs also aid in pest control by consuming insects and serve as bioindicators, reflecting the overall health of the ecosystem [29]. Collectively, these species help maintain balance in agricultural environments, reducing the need for chemical pesticides and promoting biodiversity.

Therefore, this study deeply analyzes the correlation between the feature and size of field ridges and plant and animal diversity in the paddy field ecosystem under intensive management. Three hypotheses were tested in cultivated fields as follows: (1) Wider field ridges harbor greater plant diversity; (2) higher plant diversity fosters greater animal diversity; and (3) divergent plant communities foster unique different animal communities. This research not only aids in effective ecosystem management and conservation but also enhances our understanding of how field ridges contribute to ecological stability and biodiversity in paddy field landscapes.

## 2. Materials and Methods

### 2.1. Study Area

The study area was located in the western area of Chengdu Plain, China. The region is characterized by its flat terrain, dominated by rice cultivation. It boasts a vast demonstration zone for well-facilitated paddy fields spanning thousands of acres since the well-facilitated construction measures were launched in the early 2010s in China [30], presenting a relatively simplistic landscape. The area experiences a subtropical monsoon climate with distinct wet and dry seasons. Salient features of its climate include its mild temperatures, clear distinction of the four seasons, a long frost-free period averaging around 280 days annually, an average annual temperature of 16 °C, an average annual sunlight duration of 927.5 h, and an average annual precipitation of 1114.7 mm, creating favorable conditions for crop cultivation.

**Figure 1:**
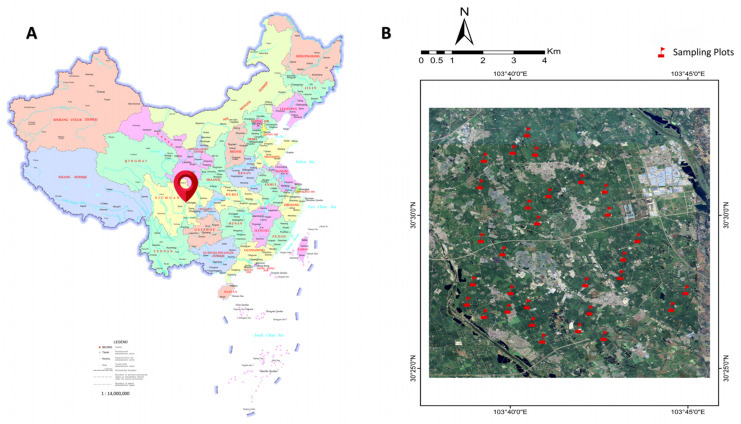
Location of study areas (**A**). The red note indicates the location of each sampling plot (**B**) in the study areas. The map of China is a 1400 standard map drawn by the Ministry of Natural Resources of the PRC, and the approval number is GS [2022] No. 4308.

Thirty field ridges of paddy fields around the study area were selected randomly, and both sides of the field ridges were planted with rice. The smallest distance between any two field ridges was 1 km to ensure the independence of biodiversity sampling (Figure 1).

### 2.2. Biodiversity Survey

The sampling plot of the plant survey was located in the center of the field ridges; its length was according to its width, and the total sampling area was 10 m^2^ for each field ridge. An estimation method was utilized to record the total vegetative coverage of vascular plant communities and also the coverage of each plant species. The coverage level was estimated by the Brann-Blanquet method [31]. Surveys were conducted once in September 2020 and others twice in July and September 2021. Bird surveys primarily adopted the point count method, utilizing binoculars for observation. This survey was conducted under a wind speed of less than 4 m/s and clear sky conditions, specifically between 7:00 and 10:00 in the morning and 15:00 and 18:00 in the afternoon, when bird activity was active. Bird species seen or heard within a 50 m radius from the central point of the field ridge were recorded. Each observation lasted for 15 min, with two observation sessions in July and September 2021.

Arthropods within the rice vegetation layer were sampled using an aspirator modified from a compound leaf blower-vac. This method better prevents damage to the arthropods during the suction process, ensuring the integrity of the specimens [32]. Six sampling sessions were conducted during the rice seedling phase, which continued until the rice harvest period (about from June to September) for both 2020 and 2021 annually. In each sampling session, sample points were established on both sides of the field ridge, with four points per side. These points were positioned more than 1 m away from the field ridges and maintained a distance of at least 2 m among them. A bottomless plastic bucket (base diameter of 40 cm, height of 40 cm, and volume of approximately 50 L) was swiftly placed over 2–4 rice plants at each sampling point. The modified aspirator was then inserted into the bucket to suction for 2 min. Specimens were preserved in 80% alcohol and returned to the laboratory for taxonomic identification.

The ground pitfall trap was employed for sampling epigeal arthropods; however, some frogs also fell into the cup. Six pitfall traps were set up along the center of field ridges, spaced 2 m apart. Each trap had an opening diameter of 8 cm and a capacity of approximately 450 mL. They were filled with 2/3 saturated saltwater solution and a few drops of detergent. A rain shield was positioned 5 cm above each trap. The solution was exchanged every week, and the arthropods and frogs within it were collected. In 2020, six consecutive sampling weeks were conducted from mid-July to early September. After two weeks of continuous sampling, sampling continued after an interval of two weeks, totaling six sampling weeks from late May to early September 2021.

### 2.3. Statistical Analysis

We initially focused on the effect of total vegetation cover and the ridge width of the field ridge on plant and animal diversity. We applied an arcsine transformation combined with the square root function for the cover of each plant species; this transformation is often used to normalize percentage data by first taking the square root of the data and then applying the arcsine transform to reduce the effect of extreme values and improve the symmetry of the data distribution. The specific transformation formula is as follows: $y=acsin\left(\sqrt{x}\right)$, *x* represents the original data, and *y* is the transformed data. Taking into account two plant surveys conducted in 2021, these survey data were combined for statistical analysis. For each plot, if a plant was recorded in both surveys, the maximum coverage of that plant in both surveys was selected as the plant data for that plot. This approach ensures that the plant cover data for each plot best reflects the actual situation. The PAST software, https://past.en.lo4d.com, accessed on 15 February 2024 [33] was utilized to calculate alpha-diversity indices, such as species richness, the Shannon index, the evenness index (just for plants), abundance, and Fisher’s alpha of each animal species.

To further elucidate the relationships between plant and animal diversity, Pearson’s correlation coefficients of alpha-diversity indices between different taxons were computed. The Pearson correlation coefficient is used to evaluate the linear correlation between two variables and is calculated as $r=\frac{\sum \left(x_{i}-\bar{x}\right)\left(y_{i}-\bar{y}\right)}{\sqrt{\sum {\left(x_{i}-\bar{x}\right)}^{2}\sum {\left(y_{i}-\bar{y}\right)}^{2}}}$, where $x_{i}$ and $y_{i}$ are the observed values of the two variables respectively, and $\bar{x}$ and $\bar{y}$ are their mean values. In order to quantify the relative importance of field ridge structural attributes and plant diversity on the species composition of animal communities, we employed tripartite redundancy analysis (tb-RDA) [34]. Prior to tb-RDA, the data pertaining to animal counts underwent “Hellinger” transformation to ensure a linear response and mitigate potential biases from low-abundance species. The formula for the Hellinger transformation:$\;y_{ij}=\frac{\sqrt{x_{ij}}}{\sqrt{\sum_{j}x_{ij}}}$, $x_{ij}$ includes the original count data of species *j* in the *i* plot, where $y_{ij}$ is the transformed data.

To delve into the relationship between plant and animal species composition on field ridges, i.e., whether a specific plant species corresponds to a specific animal species, the Mantel test was used based on the Bray–Curtis dissimilarity matrix. The formula for the Bray–Curtis dissimilarity matrix is $D_{ij}=\frac{\sum \left|x_{ik}-x_{jk}\right|}{\sum \left(x_{ik}+x_{jk}\right)}$, where $x_{ik}$ and $x_{jk}$ are the abundance of *k* species in plots *i* and *j*. The Mantel test is used to evaluate the correlation between two distance matrices, and its statistic is the Pearson correlation coefficient between the matrices. For plant canopy cover percentages and animal abundance, given their different scales, logarithmic transformations were first applied. Subsequent normalization was conducted to mitigate skewness and ensure that both datasets operated on the same scale. Furthermore, we embraced co-correspondence analysis (COCA) to identify species pairs with potential specific correlations between plant and animal communities, aiming to determine if an overarching correlation exists. COCA is a multivariate analysis method designed to reveal common patterns of change between the two biomes [25].

At last, to delve deeper into the variation in species composition within the same plots across different years, non-metric multidimensional scaling (NMDS), also based on the Bray–Curtis dissimilarity matrix of plants or spiders, was employed [35].

The analysis (Figure S1) was carried out in R 4.04 [36].

## 3. Results

A total of 90 plant species were surveyed in both years. In 2020, 50 species were identified, while in 2021, 71 species were documented. In individual sampling sites, the maximum number of plant species observed was 28, while the minimum was 3.

In 2020, a total of 7110 spiders were captured by pitfall trap and suction methods, of which 3615 were adults, including 14 families, 23 genera, and 42 species. In 2021, 5743 spider individuals were collected, with 4004 adults representing 12 families, 35 genera, and 44 species.

A total of 1989 individual frogs were identified in 2020, and 2026 individual frogs were identified in 2021, only belonging to 2 species. Additionally, 26 species of carabids were identified across 701 individuals, with 2125 birds spanning 48 species, and a total of 3987 rice plant hoppers were captured in 2021.

### 3.1. Pearson Correlation Analysis

There were no significant relationships between the ridge width (RW) and plant diversity. Moreover, RW showed a significant negative correlation with the spider’s Shannon diversity (TC3) (r = −0.413, *p* < 0.05) in 2020. But RW demonstrated a strong positive correlation with spider species richness (TC1) (r = 0.448, *p* < 0.05) and spider abundance (TC2) (r = 0.418, *p* < 0.05) (Table 1B). In 2021, Shannon diversity for birds (B3) exhibited a negative association with RW (r = −0.408, *p* < 0.05).

In 2020, there was a significant negative correlation between the spider’s Fisher’s α diversity and plant evenness (PE) (r = −0.376, *p* < 0.05) (Table 1A). Furthermore, spiders’ Shannon diversity (TC3) showed a negative correlation with plant species richness (P1) (r = −0.409, *p* < 0.05) in 2021, while spider abundance (TC2) displayed a robust positive correlation with plant species richness (P1) (r = 0.468, *p* < 0.01). Both carabid Shannon (Ca3) and Fisher’s α diversities (Ca4) presented marked negative correlations with plant evenness (PE) (r = −0.451, *p* < 0.05; r = −0.472, *p* < 0.01) in 2021. Besides these, we did not find any other significant relationships. Even considering the difference in the sampling method for spiders, there was still a lack of significant relationships among different taxa (Table S1).

### 3.2. RDA Analysis

**Figure 2:**
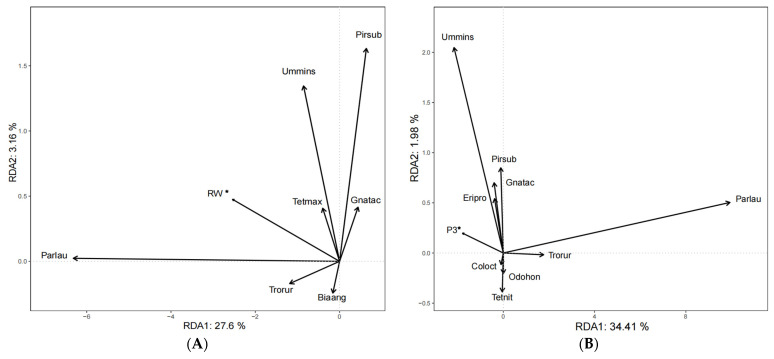
tb-RDA based on spider composition, plant diversity, and shape attributes of paddy field ridges in 2020 (**A**) and 2021 (**B**) (for a detailed list of spider species, please refer to Table S2B).

The tb-RDA analysis only underscored the significant influence of RW (ridge width) on spider composition (Figure 2A,B). Specifically, the three spider species *Pardosa laura* (*Parlau*), *Ummeliata insecticeps* (*Ummins*), and *Pirata subparaticus* (*Pirsub*) showed a high sensitivity to the width of field ridges during 2020. Contrary to the observations made in 2020, the data from 2021 indicated that the plant Shannon index (P3) plays a significant role in influencing spider composition. For example, the spider species *Ummins*, *Pirsub*, *Gnathonarium taczanowskii* (*Gnatac*), and *Erigone prominens* (*Eripro*) showed a positive correlation with P3, while *Parlau*, *Trochosa ruricola* (*Trorur*) were negatively correlated.

### 3.3. Dissimilarity of Species composition

Only a significant association was discerned between plants and frogs in 2020 (Table 2A) based on the Mantel test (r = 0.2713, *p* < 0.05), but only two frog species were used in our data. Nevertheless, there were no significant associations among the taxa in 2021 (Table 2B).

### 3.4. Co-Correspondence Analysis

Only the relationship between carabids or spiders and plants data in 2021 was significant within the permutation test by CoCA analysis, while no significance was observed in the analysis results of 2020.

**Figure 3:**
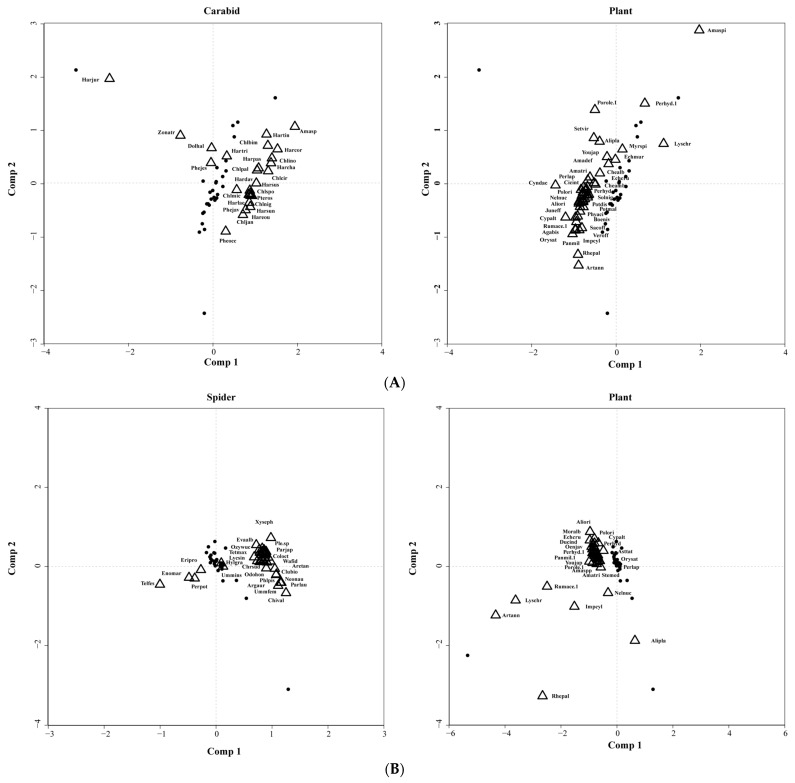
Biplot based on predictive co-correspondence analysis of carabids (**A**) and spiders (**B**) against plants. The species (triangles) are positioned in the graph according to their loadings with respect to normalized site scores (triangles) derived from plants, and black dots represent sampling plots.

From the biplots, it is evident that the majority of carabid species and plants are congregated in the same region of the plot (Figure 3A), which means that there is no specific correlation. The biplot for carabids against plants accounted for 12.9% and 13.18% of the total variation, respectively. Only a select few carabid species showed noticeable correlations with specific plants such as *Pheropsophus occipitalis* and *Rheum palmatum*, *Harpalus pastor*, and *Echinochloa muricata*. Similarly, the biplot between spiders and plants elucidated 12.88% and 7.82% of the total variation. Predominant spider species and plants reside within the same region, with merely a handful of spider species exhibiting significant correlations with certain plants, such as *Chinattus validus* and *Nelumbo nucifera*, *Rumex acetosa*, and *Gnathonarium taczanowskii* (Figure 3B).

### 3.5. NMDS

**Figure 4:**
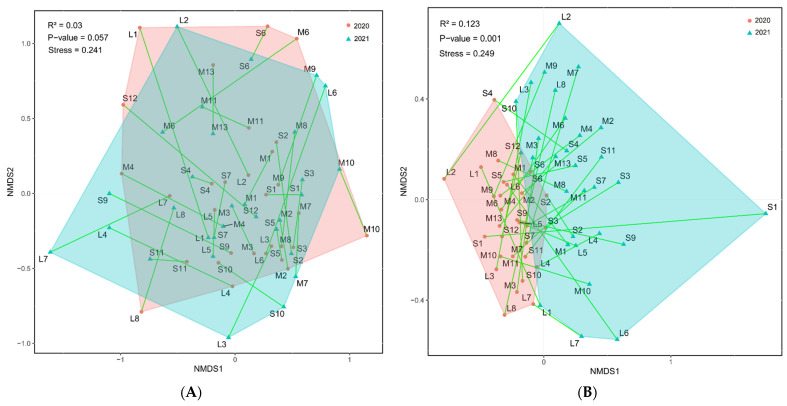
Non-metric multidimensional scaling (NMDS) based on the Bray–Curtis Dissimilarity matrix of plants (**A**) and spiders (**B**). The two sampling years for the same plots were connected by a thin line. Points in the same background color range represent plots sampled in the same year.

The NMDS analyses showed there were significant differences in spider species composition between 2020 and 2021, which is approximately significant for plants (Figure 4A,B). Additionally, specific pairwise distances of the same plot between 2020 and 2021 were computed, and the average distance across all plots in the same year was also determined. There was no distinct difference between them. Even considering the difference in sampling method for spiders, significant differences were also present (Figure S2).

## 4. Discussion

Different from the results of other studies, we did not find strong connections or interactions between plant and animal communities in the field ridges of the paddy landscape in the western Chengdu Plain, China. Our results indicate the inconsistent spatial distribution of different plant and animal communities in paddy ecosystems. This implies that a high level of diversity in one taxon does not necessarily indicate a similar level of diversity in other taxa, including those that have direct trophic relationships [24]. If different taxa are differentially susceptible to extrinsic factors, such as climate, temperature, nutrients, human disturbance, etc., it may also lead to a lack of highly correlated diversity levels between different taxa [37,38].

Studies have shown that more than 63% of arthropod species within intensively managed farmland depend on semi-natural habitats [28,39]. Semi-natural habitats are treated with lower quantities of agrochemicals than adjacent cultivated fields and benefit biodiversity [28]. In general, linear semi-natural habitats can also provide habitat resources (including nesting or overwintering sites or shelter habitats during crop management) or food resources and enrich species richness in farmland [40,41,42,43]. Therefore, as one of the most important linear habitats in the paddy field ecosystem, the physical (width, etc.) and chemical properties of the ridge have the greatest effect on plant and animal diversity [39]. In addition, as one semi-natural habitat type, the ridge also provides a better habitat for plants and animals than the interior of the field [44,45]. Under suitable environmental conditions, the greater the habitat area, the higher the biodiversity [46]; thus, a wider ridge represents a larger growth, development, and activity area, a more hidden space for organisms, and should have a higher diversity of plants and animals [47]. However, in our two-year data survey, we did not find that ridge width had a significant effect on plant diversity, even when significantly negatively correlated with spider and bird diversity. This may be because the original soil structure and environmental conditions of the ridge changed after large-scale land consolidation in 2013, and the plants that originally grew on the ridge no longer existed. At the same time, due to long-term farming and intensive management [38,48], the reconstruction speed of the ridge was slow, and there was not enough time to form a stable plant community, nor could it provide a stable habitat space for animals [49,50]. Some dominant species, such as *Digitaria sanguinalis*, *Leptochloa chinensis*, *Echinochloa crus-galli*, and invasive alien species *Alternanthera philoxeroides* have strong adaptability to this environment, strong growth, and relatively vigorous growth. The interspecific competitiveness is greater, which inhibits the growth of other plants and leads to the homogeneity of plants growing on the field ridge, which is not suitable for animals to forage and roost in. In addition, when the field is too wide, it forms a relatively open space, and birds can be easy to find, increasing their risk of predation by the enemy [46,51]. However, we also found from the results of 2021 that the width of the ridge showed a significant positive correlation with spider diversity, which can also indicate that a more open living space is indeed conducive to spider diversity. In addition, there may be an inconsistent correlation between the width of the ridge and the same biological group in different periods. Therefore, a wider ridge in the paddy field does not mean higher biodiversity, which is related to the specific needs of different biological groups, historical factors, and the microenvironment of the habitat.

Traditional theories and many previous studies hold that plant diversity is positively correlated with animal diversity in an ecosystem; higher plant diversity can provide suitable habitat space and rich food sources for different species of animals, thus attracting a wide variety of animals to settle [22,52,53,54]. Therefore, an ecosystem with a higher plant diversity is often accompanied by a higher animal diversity [54]. However, our results show an inconsistent correlation between animal diversity and plant diversity. This suggests that the correlation between plants and animals in unstable semi-natural habitats is not simply positive. This inconsistency can be explained for the following reasons: (1) the temporal framework of our botanical and zoological sampling was confined to August and September. The Spillover Effect Hypothesis posits that during the booting and mature stage of rice cultivation [55], the paddy ecosystem becomes a trove of nutritional resources for animal communities such as spiders, carabids, and avian species [56]. As a result, the interior of the field is more attractive to organisms than the linear plant community at the field ridge. During this period, farmland crops grow well, plants are rich in nutrients, there are more plant-eating insects, and more natural enemies enter the interior of the field. (2) The intricate interplay between vegetation structure and spider diversity has been the focal point of many investigations. The prevailing literature emphasizes the positive correlation between vegetation stature and spider diversity [53,57]. For web-building spiders, higher plant height can provide more space for weaving webs, which is more conducive to roosting and hunting. In addition, the higher the height of the plant, the better shelter it provides for the hunting spider [58,59]. Our analysis has revealed that the canopy density of plant species such as *Echinochloa crus-galli* (*Echcru*), *Euphorbia lathyrism* (*Euplat*), and *Ludwigia epilobioides* (*Ludepi*) exert a positive influence on the abundance of *Lycosidae*. Plant height has a greater effect on arthropods than plant diversity [58,59]. These plant communities can attain growth higher than 1m, offering an expansive ecological niche for hunting spiders and engendering conducive locales for web-building spiders [58,59]. So even if plant diversity is low, but the overall height of vegetation is higher, the spider diversity is also higher. In addition, the fields ridge with *Amaranthus viridis* (*Amavir*) and *Acalypha australis* (*Acaaus*) have a higher relative diversity index for spiders because both *Amavir* and *Acaaus* are the higher plant species. This suggests that the presence of plants with an appropriately high vegetation community can provide the best habitat for spiders, which is conducive to the improvement of spider diversity [60]. (3) Arthropod groups have different responses to different plant species on the field ridge [46], so even though the diversity of plants on the ridge is high, most plant species may not be suitable for foraging and roosting, resulting in a low diversity of spiders and carabids. At the same time, even though the plant diversity on the ridge is low, some spiders have adapted to the environment of common plant species, so the abundance of some spiders is higher [39,61]. Therefore, the overall plant diversity on the ridge is not a good representation of the characteristics and heterogeneity of the whole plant community, and more indicators are needed to measure the plant community on the ridge, such as the height and differences in different functional groups, to establish a more accurate relationship with animal communities.

From our investigation into the cross-taxon congruence in species turnover patterns, most results indicate no significant associations. It means that the second hypothesis that the community compositions of divergent plant species correspond to distinct animal communities may not be true in our study. To elucidate this observed discrepancy, several reasons should be considered as follows: (1) The observed significant positive correlation between the species composition of frogs and plants in 2020 may be due to the different plant communities on the ridge that can provide different habitats and shelter for the two species of frog. But the subsequent lack of significance in 2021 may reflect the complex and dynamic interplays within the paddy field ridge compared to other stable ecosystems, such as forests and wetlands [29,62]. This variation could be attributed to changes in ecological conditions over the year, such as climatic differences, alterations in hydrological conditions, or shifts in habitat quality, which, in turn, affected the interdependent relationship between plant and frog communities. Additionally, intrinsic dynamics within these communities, such as changes in population density, adjustments in competitive interactions, and fluctuations in disease or predation pressures, might have influenced the relationship between them [63]. (2) There is a lack of a direct relationship between vascular plants and our surveyed animal communities. For example, birds may primarily operate within certain trophic levels, such as insectivores (frogs, etc.), which reduces their direct reliance on the plant-based food web of the field ridge [63,64,65]. Spider and carabid species could follow different trophic pathways or have a diverse range of diets [23,66]. Thus, this may be the main reason for the lack of consistency between them.

Another probable reason for no species-level match or direct correspondence among different taxa could be the serious degrees of damage that field ridges experience during mechanical crushing and destroying in the ploughing, sowing, and harvesting phases [67,68]. Especially for winter wheat planting, many fields are also covered with wheat and are mechanically harvested in early summer. In addition, some drones spray pesticides indiscriminately on the ridge. As a result, the growth of vascular plants becomes restricted or even devastated, and the habitats and activity zones for various animal communities are likewise compromised. This led to a significant reduction or even disappearance of plant species and arthropod communities on the ridges of fields within certain periods of our study area. However, post-harvest and sowing, the ecosystem tends to recuperate, with new plants emerging on the ridges and attracting new animals. Over time, this leads to the ecosystem of the paddy fields and ridges perpetually oscillating between phases of “destruction and reconstruction”. The biotic components of this ecosystem remain in a state of constant random assembly, preventing stable relationships from forming among species over extended periods. This dynamic results in unstable interspecific relationships [65]. To further validate our deduction, the NMDS indicates that both plant and spider communities, even in the same plots, experienced pronounced shifts from 2020 to 2021, with distinct differences in community structure composition (Figure 4). Additionally, the CoCA results predominantly showed species clustering in similar regions (indicating no correlation), with only a few species of spiders and carabids possibly having a corresponding relationship with plants. This lends credence to our hypothesis, further emphasizing that the field ridges in intensified agricultural landscapes fail to uphold and maintain biodiversity.

In this study, two years of experimental observations explicitly pointed out that the role of field ridges in biodiversity conservation may be limited in the intensively managed paddy landscape. Specifically, the diversity of spiders, birds, and carabids mostly shows negative correlations with the plant diversity of field ridges. These findings form a stark contrast to the general belief in previous studies that field ridges with a high plant diversity can increase or maintain animal diversity [28,39,59], further emphasizing that relying solely on field ridges to protect biodiversity in modern agricultural practices may be insufficient and unsustainable. In particular, unstable and repeatedly changing field ridges cannot provide high-quality plant communities and, thus, provide suitable habitats for corresponding animal communities. Some previous studies have been inclined to the belief that the weeds on field ridges can become hotbeds for diseases, pests, and weed infestations [69,70,71], posing a competitive threat to adjacent crops for essential resources such as nutrients, light, and water [17,18,72,73]. Consequently, farmers often resort to the application of herbicides or manual weeding to control weed growth [70,71,74]. Mechanized production, especially winter wheat cultivation, tends to encroach on the ridge, leading to the destruction of wild plants on the ridge. These practices, however, result in a constantly fluctuating plant community on the field ridges, preventing the formation of stable communities and, in turn, failing to support corresponding animal populations. The species of plants and animals found are typical “generalists” well-adapted to the agricultural environment, such as *Harpalus sinicus*, *Pardosa laura Karsch*, *Passer*, etc., with a notable absence of high-value “specialists” that might otherwise thrive in more stable or diverse habitats, such as *Trigonognatha cuprescens*, *Xysticus*, *Chloris sinica*, etc. [26].

Simultaneously, these observations also show that even if the plant diversity on field ridges varies in different years, these variations do not lead to a corresponding increase or improvement in animal diversity. This further highlights that there may not be a simple positive correlation between plant and animal diversity. These findings not only stress the need to reconsider the role and effects of field ridges and other land-use types in biodiversity conservation in agricultural ecosystems but also suggest that more comprehensive and diversified biodiversity conservation strategies may be needed for the more effective protection of biodiversity. For example, promoting structurally diverse farmlands (e.g., such structures as grassland buffer strips, small patches of uncropped land, woody hedges and corridors, flower-rich habitats, and patches of open soil) may contribute substantially to ecosystem functioning and ecosystem services [28,75].

This study covered 30 field ridges in the paddy landscape with well-facilitated measures, including six taxa with different trophic levels, and analyzed the correlation between plant and animal diversity from taxa to specific species through the perspective of α diversity and β diversity at two different scales of biodiversity. The degree of congruence of correlations among different taxa is also discussed. There are also some defects and inadequacies in this study. We recorded the species and number of birds seen and heard within fifty meters of the ridge center, including the birds that just flew over our sampling plot. Frogs were caught by the ground pitfall trap, not the fence trap method, so only two frog species were surveyed. The result does not represent the total frog community. The suction method of spiders was carried out on both sides of the ridge within the paddy fields but not in the ridge-like ground pitfall traps. However, our survey is consistent in all thirty field ridges, and there is no sampling bias. Thus, these results are credible overall.

## 5. Conclusions

In the intensive paddy landscape of Chengdu plain, due to mechanized ploughing, sowing, harvesting, and weeding, there was no strong correlation between plant diversity and animal diversity in the field ridge. The annual periodic destruction and reconstruction of plant and animal communities cannot support stable plant communities and corresponding animal communities in field ridges. The plant diversity of paddy field ridges under intensive management is not enough to support animal diversity. We observed that despite the intrinsic ecological potential of the field ridges, they suffer significant degradation due to human interventions, particularly during critical periods such as sowing and harvesting. This degradation disrupts the sustained congruence between plant species on the ridges and the residing animal communities in the farmlands; thereby, the capacity of the ridges to preserve and enhance farmland biodiversity is low. The recurring cycle of “destruction-reconstruction” highlights the inherent challenges in fostering stable interspecies relationships within such contexts. Therefore, maintaining the stability and long-term plant community of the ridge and avoiding human interference and destruction are the most important factors for protecting the farmland biodiversity of the whole paddy landscape.

There is an urgent call for sustainable agricultural practices, with an emphasis on protective measures for semi-natural habitats like field ridges. A more profound exploration into farmland biodiversity is essential to both bolster its conservation and further the agenda of sustainable agricultural progression.

## Figures and Tables

**Table 1:** Correlation between trait (width), plant and animal diversity in field ridges in 2020 (A) and 2021 (B).

**(A)**									
	**RW**	**P1**	**PE**	**TC1**	**TC2**	**TC3**	**B3**	**Ca3**	**Ca4**
RW	1								
P1	0.138	1							
PE	0.001	0.111	1						
TC1	0.448 *	0.167	−0.115	1					
TC2	0.418 *	0.468 **	0.019	0.614 **	1				
TC3	0.009	−0.409 *	0.062	0.265	−0.48 **	1			
B3	−0.408 *	−0.025	−0.083	−0.031	−0.263	0.104	1		
Ca3	−0.027	0.303	−0.451 *	0.349	0.227	−0.018	0.308	1	
Ca4	−0.182	0.12	−0.472 **	0.019	0.042	−0.085	0.259	0.708 **	1
**(B)**									
	**RW**	**RC**	**P1**	**P3**	**PE**	**TC1**	**TC2**	**TC3**	**TC4**
RW	1								
RC	0.081	1							
P1	−0.096	0.076	1						
P3	−0.05	−0.035	0.924 **	1					
PE	−0.282	−0.235	0.302	0.569 **	1				
TC1	0.063	0.169	−0.162	−0.235	−0.253	1			
TC2	0.268	0.09	0.035	0.079	0.135	0.544 **	1		
TC3	−0.413 *	0.1	0.008	−0.128	−0.224	0.651 **	0.03	1	
TC4	−0.204	0.018	−0.17	−0.29	−0.376 *	0.667 **	−0.213	0.709	1

RC = ridge coverage, RW = ridge width, P = plant, TC = spider, B = bird, Ca = carabid, 1 = species richness, 2 = species abundance, 3 = Shannon, and 4 = Fisher’s α, PE = plant eveness. * ≤ 0.05; ** ≤ 0.01, all bold numbers represent the significant correlation.

**Table 2:** The result of the Mantel test between plant and animal species composition.

**(A)**					
	**Plant**	**Spider**	**Frog**		
Plant	1				
Spider	0.084	1			
Frog	0.177 *	0.028	1		
**(B)**					
	**Plant**	**Spider**	**Carabid**	**Bird**	**Frog**
Plant	1				
Spider	−0.013	1			
Carabid	0.081	0.004	1		
Bird	−0.034	0.016	−0.026	1	
Frog	−0.024	−0.02	−0.113	−0.236	1

* ≤ 0.05, all bold numbers represent the significant correlation.

## Data Availability

The original contributions presented in the study are included in the article/Supplementary Material, further inquiries can be directed to the corresponding author.

## Supplementary Materials

The following supporting information can be downloaded at https://www.mdpi.com/article/10.3390/plants13121680/s1, Table S1. Correlation between trait (width), plant and animal diversity in field ridges in 2020(A) and 2021 (B), based on different sampling method. Table S2. Species abbreviation. Figure S1. Flow chart of data processing and correlation analysis. Figure S2. Non-metric multidimensional scaling (NMDS) is based on the Bray–Curtis dissimilarity matrix of different sampling methods for the spider (A) ground pitfall method and (B) suction method. The two sampling years for the same plots are connected by a thin line. Points in the same background color range represent plots sampled in the same year.

## References

[1] Ramankutty N., Mehrabi Z., Waha K., Jarvis L., Kremen C., Herrero M., Rieseberg L.H. (2018). Trends in Global Agricultural Land Use: Implications for Environmental Health and Food Security. Annu. Rev. Plant Biol..

[2] Tilman D., Balzer C., Hill J., Befort B.L. (2011). Global Food Demand and the Sustainable Intensification of Agriculture. Proc. Natl. Acad. Sci. USA.

[3] Gong S., Zhou X., Zhu X., Huo J., Faghihinia M., Li B., Zou Y. (2023). Organic Rice Cultivation Enhances the Diversity of Above-Ground Arthropods but Not below-Ground Soil Eukaryotes. Agric. Ecosyst. Environ..

[4] Seppelt R., Arndt C., Beckmann M., Martin E.A., Hertel T.W. (2021). Deciphering the Biodiversity-Production Mutualism in the Global Food Security Debate: (Trends in Ecology and Evolution 35, 1011–1020). Trends Ecol. Evol..

[5] Lewandowski A.S., Noss R.F., Parsons D.R. (2010). The Effectiveness of Surrogate Taxa for the Representation of Biodiversity. Conserv. Biol..

[6] Haddad N.M., Crutsinger G.M., Gross K., Haarstad J., Knops J.M.H., Tilman D. (2009). Plant Species Loss Decreases Arthropod Diversity and Shifts Trophic Structure. Ecol. Lett..

[7] Castagneyrol B., Jactel H. (2012). Unraveling Plant–Animal Diversity Relationships: A Meta-Regression Analysis. Ecology.

[8] Albert G., Gauzens B., Ryser R., Thébault E., Wang S., Brose U. (2023). Animal Movement and Plant Space-Use Drive Plant Diversity-Productivity Relationships. Authorea.

[9] Wang M., Christoph Axmacher J., Yu Z., Zhang X., Duan M., Wu P., Zou Y., Liu Y. (2021). Perennial Crops Can Complement Semi-Natural Habitats in Enhancing Ground Beetle (Coleoptera: Carabidae) Diversity in Agricultural Landscapes. Ecol. Indic..

[10] Marshall E.J.P., Moonen A.C. (2002). Field Margins in Northern Europe: Their Functions and Interactions with Agriculture. Agric. Ecosyst. Environ..

[11] Liu Y., Yu Z., Gu W., Axmacher J.C. (2006). Diversity of Carabids (Coleoptera, Carabidae) in the Desalinized Agricultural Landscape of Quzhou County, China. Agric. Ecosyst. Environ..

[12] Luo Y., Fu H., Traore S. (2014). Biodiversity Conservation in Rice Paddies in China: Toward Ecological Sustainability. Sustainability.

[13] Sawicka B., Krochmal-Marczak B., Barbaś P., Pszczółkowski P., Ćwintal M. (2020). Biodiversity of Weeds in Fields of Grain in South-Eastern Poland. Agriculture.

[14] Nelson K.S., Burchfield E.K. (2021). Landscape Complexity and US Crop Production. Nat. Food.

[15] Asseng S., Pannell D.J. (2013). Adapting Dryland Agriculture to Climate Change: Farming Implications and Research and Development Needs in Western Australia. Clim. Change.

[16] Zumpf C., Quinn J., Cacho J., Grasse N., Negri M.C., Lee D. (2021). Invertebrate and Plant Community Diversity of an Illinois Corn–Soybean Field with Integrated Shrub Willow Bioenergy Buffers. Sustainability.

[17] Osawa T., Nishida T., Oka T. (2020). Paddy Fields Located in Water Storage Zones Could Take over the Wetland Plant Community. Sci. Rep..

[18] Zhuang Y., Liu H., Zhang L., Li S. (2020). Research Perspectives on Paddy Field Systems: Ecological Functions and Environmental Impacts. Int. J. Agric. Sustain..

[19] Natuhara Y. (2013). Ecosystem Services by Paddy Fields as Substitutes of Natural Wetlands in Japan. Ecol. Eng..

[20] Hamano M., Shiozawa S., Yamamoto S., Suzuki N., Kitaki Y., Watanabe O. (2023). Development of a Method for Detecting the Planting and Ridge Areas in Paddy Fields Using AI, GIS, and Precise DEM. Precis. Agric..

[21] Liu Y., Duan M., Zhang X., Zhang X., Yu Z., Axmacher J.C. (2015). Effects of Plant Diversity, Habitat and Agricultural Landscape Structure on the Functional Diversity of Carabid Assemblages in the North China Plain. Insect Conserv. Divers..

[22] Li X., Liu Y., Duan M., Yu Z., Axmacher J.C. (2018). Different Response Patterns of Epigaeic Spiders and Carabid Beetles to Varying Environmental Conditions in Fields and Semi-Natural Habitats of an Intensively Cultivated Agricultural Landscape. Agric. Ecosyst. Environ..

[23] Topping C.J. (1993). Behavioural Responses of Three Linyphiid Spiders to Pitfall Traps. Entomol. Exp. Appl..

[24] Duan M., Liu Y., Yu Z., Baudry J., Li L., Wang C., Axmacher J.C. (2016). Disentangling Effects of Abiotic Factors and Biotic Interactions on Cross-Taxon Congruence in Species Turnover Patterns of Plants, Moths and Beetles. Sci. Rep..

[25] ter Braak C.J.F., Schaffers A.P. (2004). Co-Correspondence Analysis: A New Ordination Method to Relate Two Community Compositions. Ecology.

[26] Duan M., Hu W., Liu Y., Yu Z., Li X., Wu P., Zhang F., Shi H., Baudry J. (2019). The Influence of Landscape Alterations on Changes in Ground Beetle (Carabidae) and Spider (Araneae) Functional Groups between 1995 and 2013 in an Urban Fringe of China. Sci. Total Environ..

[27] Gallé R., Geppert C., Földesi R., Tscharntke T., Batáry P. (2020). Arthropod Functional Traits Shaped by Landscape-Scale Field Size, Local Agri-Environment Schemes and Edge Effects. Basic Appl. Ecol..

[28] Šálek M., Hula V., Kipson M., Daňková R., Niedobová J., Gamero A. (2018). Bringing Diversity Back to Agriculture: Smaller Fields and Non-Crop Elements Enhance Biodiversity in Intensively Managed Arable Farmlands. Ecol. Indic..

[29] Oh H.-J., Chang K.-H., Jin M.-Y., Suh J.-M., Yoon J.-D., Shin K.-H., Park S.-G., Chang M.-H. (2021). Trophic Ecology of Endangered Gold-Spotted Pond Frog in Ecological Wetland Park and Rice Paddy Habitats. Animals.

[30] Sun C., Yang H., Han D., Hao C., Li H. (2022). National High Standard Farmland Construction Situation and Development Strategy. China Agric. Sci. Technol. Her..

[31] Willner W., Faber-Langendoen D. (2021). Braun-Blanquet Meets EcoVeg: A Formation and Division Level Classification of European Phytosociological Units. Veg. Classif. Surv..

[32] Zou Y., van Telgen M.D., Chen J., Xiao H., de Kraker J., Bianchi F.J.J.A., van der Werf W. (2016). Modification and Application of a Leaf Blower-Vac for Field Sampling of Arthropods. J. Vis. Exp..

[33] Hammer Ø., Harper D.A.T., Ryan P.D. (2001). PAST: Paleontological Statistical Software Package for Education and Data Analysis. Palaeontol. Electron..

[34] Kermavnar J., Marinšek A., Eler K., Kutnar L. (2019). Evaluating Short-Term Impacts of Forest Management and Microsite Conditions on Understory Vegetation in Temperate Fir-Beech Forests: Floristic, Ecological, and Trait-Based Perspective. Forests.

[35] Jiao S., Chen W., Wang J., Du N., Li Q., Wei G. (2018). Soil Microbiomes with Distinct Assemblies through Vertical Soil Profiles Drive the Cycling of Multiple Nutrients in Reforested Ecosystems. Microbiome.

[36] R Core Team (2014). R: A Language and Environment for Statistical Computing.

[37] Richerson P.J., Lum K. (1980). Patterns of Plant Species Diversity in California: Relation to Weather and Topography. Am. Nat..

[38] Tang H., Yun W., Liu W., Sang L. (2019). Structural Changes in the Development of China’s Farmland Consolidation in 1998–2017: Changing Ideas and Future Framework. Land Use Policy.

[39] Duelli P., Obrist M.K. (2003). Regional Biodiversity in an Agricultural Landscape: The Contribution of Seminatural Habitat Islands. Basic Appl. Ecol..

[40] Bianchi F.J.J.A. (2022). From Pattern to Process: Towards Mechanistic Design Principles for Pest Suppressive Landscapes. Basic Appl. Ecol..

[41] Boetzl F.A., Krimmer E., Holzschuh A., Krauss J., Steffan-Dewenter I. (2022). Arthropod Overwintering in Agri-Environmental Scheme Flowering Fields Differs among Pollinators and Natural Enemies. Agric. Ecosyst. Environ..

[42] Poschlod P., Braun-Reichert R. (2017). Small Natural Features with Large Ecological Roles in Ancient Agricultural Landscapes of Central Europe—History, Value, Status, and Conservation. Biol. Conserv..

[43] Zou Y. (2024). Evaluating the Potential of Agri-Environmental Measures (AEM) in Mitigating Biodiversity Loss Due to Land Consolidation in China: Understanding the Function of Linear Habitats. Basic Appl. Ecol..

[44] Tscharntke T., Batáry P., Dormann C.F. (2011). Set-aside Management: How Do Succession, Sowing Patterns and Landscape Context Affect Biodiversity?. Agric. Ecosyst. Environ..

[45] Zhu P., Zheng X., Johnson A.C., Chen G., Xu H., Zhang F., Yao X., Heong K., Lu Z., Gurr G.M. (2022). Ecological Engineering for Rice Pest Suppression in China. A Review. Agron. Sustain. Dev..

[46] Knapp M., Řezáč M. (2015). Even the Smallest Non-Crop Habitat Islands Could Be Beneficial: Distribution of Carabid Beetles and Spiders in Agricultural Landscape. PLoS ONE.

[47] Chiron F., Chargé R., Julliard R., Jiguet F., Muratet A. (2014). Pesticide Doses, Landscape Structure and Their Relative Effects on Farmland Birds. Agric. Ecosyst. Environ..

[48] Yin Q., Sui X., Ye B., Zhou Y., Li C., Zou M., Zhou S. (2022). What Role Does Land Consolidation Play in the Multi-Dimensional Rural Revitalization in China? A Research Synthesis. Land Use Policy.

[49] Cizek O., Zamecnik J., Tropek R., Kocarek P., Konvicka M. (2012). Diversification of Mowing Regime Increases Arthropods Diversity in Species-Poor Cultural Hay Meadows. J. Insect Conserv..

[50] Konvicka M., Benes J., Cizek O., Kopecek F., Konvicka O., Vitaz L. (2008). How Too Much Care Kills Species: Grassland Reserves, Agri-Environmental Schemes and Extinction of *Colias myrmidone* (Lepidoptera: Pieridae) from Its Former Stronghold. J. Insect Conserv..

[51] Billeter R., Liira J., Bailey D., Bugter R., Arens P., Augenstein I., Aviron S., Baudry J., Bukacek R., Burel F. (2008). Indicators for Biodiversity in Agricultural Landscapes: A Pan-European Study. J. Appl. Ecol..

[52] Rivers A.N., Mullen C.A., Barbercheck M.E. (2018). Cover Crop Species and Management Influence Predatory Arthropods and Predation in an Organically Managed, Reduced-Tillage Cropping System. Environ. Entomol..

[53] Koricheva J., Hayes D. (2018). The Relative Importance of Plant Intraspecific Diversity in Structuring Arthropod Communities: A Meta-analysis. Funct. Ecol..

[54] Jurjanz S., Plantureux S., Vivier A. (2019). Valoriser La Diversité Végétale Par La Diversité Animale: Utiliser Le Foin de Prairies Remarquables Pour Alimenter Les Animaux de Zoo. Fourrages.

[55] Rand T.A., Tylianakis J.M., Tscharntke T. (2006). Spillover Edge Effects: The Dispersal of Agriculturally Subsidized Insect Natural Enemies into Adjacent Natural Habitats. Ecol. Lett..

[56] Haan N.L., Zhang Y., Landis D.A. (2020). Predicting Landscape Configuration Effects on Agricultural Pest Suppression. Trends Ecol. Evol..

[57] Axmacher J.C., Liu Y., Wang C., Li L., Yu Z. (2011). Spatial α-Diversity Patterns of Diverse Insect Taxa in Northern China: Lessons for Biodiversity Conservation. Biol. Conserv..

[58] Öberg S. (2007). Diversity of Spiders after Spring Sowing? Influence of Farming System and Habitat Type. J. Appl. Entomol..

[59] Batáry P., Holzschuh A., Orci K.M., Samu F., Tscharntke T. (2012). Responses of Plant, Insect and Spider Biodiversity to Local and Landscape Scale Management Intensity in Cereal Crops and Grasslands. Agric. Ecosyst. Environ..

[60] Primdahl J., Peco B., Andersen E., Schramek J., Oñate J.J. (2003). Environmental Effects of Agri-Environmental Schemes in Western Europe. J. Environ. Manag..

[61] Campbell J.W., Milne M., Dinh B.T., Daniels J.C., Ellis J.D. (2020). Spider (Araneae) Abundance and Species Richness Comparison between Native Wildflower Plantings and Fallow Controls in Intensively Managed Agricultural Areas. Arthropod-Plant Interact..

[62] Dehling J.M., Dehling D.M. (2021). Conserving Ecological Functions of Frog Communities in Borneo Requires Diverse Forest Landscapes. Glob. Ecol. Conserv..

[63] Williams S.E., Hero J.-M. (2001). Multiple Determinants of Australian Tropical Frog Biodiversity. Biol. Conserv..

[64] Meemken E.-M., Qaim M. (2018). Organic Agriculture, Food Security, and the Environment. Annu. Rev. Resour. Econ..

[65] Tscharntke T., Clough Y., Wanger T.C., Jackson L.E., Motzke I., Perfecto I., Vandermeer J., Whitbread A.M. (2012). Special Issue Article: Advancing Environmental Conservation: Essays In Honor Of Navjot Sodhi Global Food Security, Biodiversity Conservation and the Future of Agricultural Intensification. Biol. Conserv..

[66] Newbold T., Hudson L.N., Hill S.L.L., Contu S., Lysenko I., Senior R.A., Börger L., Bennett D.J., Choimes A., Collen B. (2015). Global Effects of Land Use on Local Terrestrial Biodiversity. Nature.

[67] Clough Y., Kirchweger S., Kantelhardt J. (2020). Field Sizes and the Future of Farmland Biodiversity in European Landscapes. Conserv. Lett..

[68] Shi X., Xiao H., Luo S., Hodgson J.A., Bianchi F.J.J.A., He H., van der Werf W., Zou Y. (2021). Can Landscape Level Semi-Natural Habitat Compensate for Pollinator Biodiversity Loss Due to Farmland Consolidation?. Agric. Ecosyst. Environ..

[69] Monteiro A., Santos S. (2022). Sustainable Approach to Weed Management: The Role of Precision Weed Management. Agronomy.

[70] Jia W., Won O.J., Park K.W., Lee J.J. (2017). Efficacy of Glufosinate-Ammonium to Control Annual and Perennial Weeds on the Ridges of Paddy Fields. Res. Crops.

[71] Klein R.N., Wicks G.A., Martin A., Moomaw R.S., Roeth F.W., Wilson R.G., Jasa P.J. (1992). G92-1071 Ridge Plant Systems: Weed Control. Historical Materials from University of Nebraska-Lincoln Extension.

[72] Li W., He Z., Wu L., Liu S., Luo L., Ye X., Gao H., Ma C. (2022). Impacts of Co-Culture of Rice and Aquatic Animals on Rice Yield and Quality: A Meta-Analysis of Field Trials. Field Crops Res..

[73] Horgan F.G., Vu Q., Mundaca E.A., Crisol-Martínez E. (2022). Restoration of Rice Ecosystem Services: ‘Ecological Engineering for Pest Management’ Incentives and Practices in the Mekong Delta Region of Vietnam. Agronomy.

[74] Roschewitz I., Gabriel D., Tscharntke T., Thies C. (2005). The Effects of Landscape Complexity on Arable Weed Species Diversity in Organic and Conventional Farming. J. Appl. Ecol..

[75] Tscharntke T., Klein A.M., Kruess A., Steffan-Dewenter I., Thies C. (2005). Landscape Perspectives on Agricultural Intensification and Biodiversity—Ecosystem Service Management. Ecol. Lett..

