# Supplementary material for: The Low Congruence between Plant and Animal Diversity in Field Ridges of Intensively Managed Paddy Landscapes, China
Source: Plants (Basel). 2024 Jun 18;13(12):1680. doi: 10.3390/plants13121680 (PMC11207644; doi:10.3390/plants13121680)
Supplement: Supplementary file 1 [file plants-13-01680-s001.zip › plants-3031129-supplementary.pdf]

Table S1. Correlation between trait (width), plant and animal diversity in field ridges in 2020(A) and 2021 (B), based on different sampling method.

**(A)**								
	**RW**	**PSP**	**PE**	**GPT3**	**GPT4**	**SM1**	**SM2**	**F2**
**RW**	1							
**PSP**	0.028	1						
**PE**	-0.282	0.641	1					
**GPT3**	**-0.408***	-0.216	-0.23	1				
**GPT4**	-0.179	**-0.361***	**-0.419***	0.716	1			
**SM1**	0.01	0.009	0.111	-0.192	-0.265	1		
**SM2**	0.122	-0.087	-0.06	-0.171	-0.121	0.731	1	
**F2**	-0.151	-0.007	0.244	0.013	-0.179	-0.184	-0.097	1
**(B)**								
	**RW**	**P1**	**PE**	**GPT1**	**GPT2**	**B3**	**Ca3**	**Ca4**
**RW**	1							
**P1**	0.138	1						
**PE**	0.001	0.111	1					
**GPT1**	0.324	0.125	-0.129	1				
**GPT2**	**0.397***	**0.428***	0.065	0.599	1			
**B3**	**-0.408***	-0.025	-0.083	-0.005	-0.252	1		
**Ca3**	-0.027	0.303	**-0.451***	0.288	0.184	0.308	1	
**Ca4**	-0.182	0.12	**-0.472***	0.068	0.019	0.259	0.708	1

(RC=ridge coverage, RW=ridge width, P=plant, GPT=the ground pitfall trap method, SM=suction method, B=bird, Ca=carabid, F=frog, 1= species richness, 2=species abundance, 3=Shannon, and 4=Fisher's α. *≤0.05; **≤0.01; ***<0.001), all bold numbers represent significant.

Table S2. Species abbreviation

**(A)**	
**Abbreviation**	**Species name**
Sacoff	*Saccharum officinarum*
Nelnuc	*Nelumbo nucifera*
Solnig	*Solanum nigrum*
Echcru	*Echinochloa crus-galli*
Orysat	*Oryza sativa*
Setvir	*Setaria viridis*
Potdis	*Potamogeton distinctus*
Salcol	*Salsola collina*
Cyndac	*Cynodon dactylon*
Cypcom	*Cyperus compressus*
Perhyd	*Persicaria hydropiper*
Asttat	*Aster tataricus*
Achbid	*Achyranthes bidentata*

Amatri	*Amaranthus tricolor*
Myrspi	*Myriophyllum spicatum*
Carbre	*Carex breviculmis*
Amaspp	*Amaranthus spp.*
Amaspi	*Amaranthus spinosus*
Epiaur	*Epipremnum aureum*
Impcyl	*Imperata cylindrical*
Nelnuc	*Nelumbo nucifera*
Rumace	*Rumex acetosella*
Echmur	*Echinochloa muricata*
Panmil	*Panicum miliaceum*
Cypalt	*Cyperus alternifolius*
Rumace	*Rumex acetosa*
Potmal	*Potamogeton malaianus*
Perhyd	*Persicaria hydropiper*
Rapsat	*Raphanus sativus*
Boeniv	*Boehmeria nivea*
Stemed	*Stellaria media*
Eupper	*Eupatorium perfoliatum*
Kalind	*Kalimeris indica*
Rhepal	*Rheum palmatum*
Carhir	*Cardamine hirsuta*
Lyschr	*Lysimachia christinae*
Porole	*Portulaca oleracea*
Limaqu	*Limosella aquatica*
Artann	*Artemisia annua*
Juneff	*Juncus effusus*
Cheamb	*Chenopodium ambrosioides*
Youjap	*Youngia japonica*
Basalb	*Basella alba*
Echphy	*Echinochloa phyllopogon*
Amadef	*Amaranthus deflexus*
Censto	*Centaurea stoebe*
Phyaci	*Phytolacca acinosa*
Ophjap	*Ophiopogon japonicus*
Cendif	*Centaurea diffusa*
Bidpil	*Bidens pilosa*
Brajun	*Brassica juncea*
Chealb	*Chenopodium album*
Cicint	*Cichorium intybus*
Veroff	*Verbena officinalis*
Pteaqu	*Pteridium aquilinum*
Agabis	*Agaricus bisporus*

Achmil	*Achillea millefolium*
Porole	*Portulaca oleracea*
Panmil	*Panicum miliaceum*
Myrver	*Myriophyllum verticillatum*
Moralb	*Morus alba*
Oenjav	*Oenanthe javanica*
Ducind	*Duchesnea indica*
Gnaaff	*Gnaphalium affine*
Lepsat	*Lepidium sativum*
Perlap	*Persicaria lapathifolia*
Acotat	*Acorus tatarinowii*
Polori	*Polygonum orientale*
Branap	*Brassica napus*
Alipla	*Alisma plantago-aquatica*
Aliori	*Alisma orientale*

*Plant species abbreviation.

(B)

**Abbreviation**	**Species name**
Xyseph	*Xysticus ephippiafus Simon*
Coloct	*Coleosoma octomaculatum*
Odohon	*Odontodrassus hondoensis*
Perpot	*Perimonoides potanini*
Hylgra	*Hylyphantes graminicola*
Enojap	*Enoplognatha japonica*
Nesmog	*Nesticus mogera*
Parlau	*Pardosa laura*
Xyshed	*Xysticus hedini*
Tetnit	*Tetragnatha nitens*
Biahot	*Bianor hotingchiechi*
Trorur	*Trochosa ruricola*
Pirpirato	*Piratula piratoides*
Eripro	*Erigone prominens*
Parpse	*Pardosa pseudoannulata*
Pirsub	*Pirata subparaticus*
Phlpis	*Phlegra pisarskii*
Chival	*Chinattus validus*
Parjap	*Parasteatoda japonica*
Ummins	*Ummeliata insecticeps*
Chrsud	*Chrosiothes sudabides*
Gnatac	*Gnathonarium taczanowskii*
Arctan	*Arctosa tanakai*
Sibaur	*Sibianor aurocinctus*
Ozywuc	*Ozyptila wuchangensis*

Pirten	*Piratula tenuisetacea*
Cosasi	*Coscinida asiatica*
Ummfem	*Ummeliata feminea*
Clubio	*Clubiona corrugata*
Enomar	*Enoplognatha margarita*
Lycsin	*Lycosa sinensis*
Wafid	*Wadicosa fidelis*
Tetmax	*Tetragnatha maxillosa*
Zeldav	*Zelotes davidi*
Biamac	*Bianor maculatus*
Evaalb	*Evarcha albaria*
Argbru	*Argiope bruennichi*
Cycmon	*Cyclosa monticola*
Neonau	*Neoscona nautica*
Pirpira	*Pirata piraticus*
Myrfor	*Myrmarachne formicaria*
Argaur	*Argiope aurantia*
Tetext	*Tetragnatha extensa*
Telfes	*Telamonia festiva*

*Spider species abbreviation

(C)

Abbreviation	Species name
Phejav	*Pheropsophus javanus*
Phejes	*Pheropsophus jessoensis*
Amasp	*Amara sp*
Harsun	*Harpalus (Pseudoophonus) sinicus*
Harcor	*Harpalus (Pseudoophonus) coreanus*
Pteros	*Pterostichus*
Pheocc	*Pheropsophus occipitalis*
Dolhal	*Dolichus halensis halensis*
Chlspo	*Chlaenius spoliatus*
Chlino	*Chlaenius (Chlaeniellus) inops*
Harpas	*Harpalus (Pseudoophonus) pastor*
Chlnig	*Chlaenius (Epomis) nigricans*
Hartri	*Harpalus (Pseudoophonus) tridens*
Zonatr	*Zonopterus atratus*
Chlpal	*Chlaenius (Chlaenius) pallipes*
Chlcir	*Chlaenius (Chlaeniostenus) circumdatus*
Harcha	*Harpalus (Harpalus) chalcentus*
Hardav	*Harpalus (Pseudoophonus) davidi*
Hareou	*Harpalus (Pseudoophonus) eous*
Harlae	*Harpalus (Harpalus) laevipes*
Chlmic	*Chlaenius (Achlaenius) micans*
Chljan	*Chlaenius (Pachydinodes) janus*

Harsus	*Harpalus (Harpalus) sushenicus*
Hartin	*Harpalus (Zangoharpalus) tinctulus luteicornoides*
Chlbim	*Chlaenius (Lissauchenius) bimaculatus lynx*
Harjur	*Harpalus (Pseudoophonus) jureceki*

*Carabid species abbreviation.

(D)

**Abbreviation**	**Species name**
Hirrus	*Hirundo rustica*
Egrgar	*Egretta garzetta*
Strchi	*Streptopelia chinensis*
Ardalb	*Ardea alba*
Lansch	*Lanius schach*
Bubibi	*Bubulcus ibis*
Pasmon	*Passer montanus*
Garcan	*Garrulax canorus*
Strdec	*Streptopelia decaocto*
Pycsin	*Pycnonotus sinensis*
Acrcri	*Acridotheres cristatellus*
Trigla	*Tringa glareola*
Galgal	*Gallinago gallinago*
Nycnyc	*Nycticorax nycticorax*
Carspi	*Carduelis spinus*
Ardcin	*Ardea cinerea*
Garleu	*Garrulax leucolophus*
Strtra	*Streptopelia tranquebarica*
Prifla	*Prinia flaviventris*
Alaarv	*Alauda arvensis*
Stuser	*Sturnus sericeus*
Dicmac	*Dicrurus macrocercus*
Deldas	*Delichon dasypus*
Corbra	*Corvus brachyrhynchos*
Ardcin	*Ardea cinerea*
Galchl	*Gallinula chloropus*
Turmer	*Turdus merula*
Colliv	*Columba livia*
Oenoen	*Oenanthe oenanthe*
Carcar	*Carduelis carduelis*
Anapla	*Anas platyrhynchos*
Motalb	*Motacilla alba*
Ixosin	*Ixobrychus sinensis*
Egrgar	*Egretta garzetta*
Apuaff	*Apus affinis*
Chadub	*Charadrius dubius*

Cuccan	*Cuculus canorus*
Upuepo	*Upupa epops*
Cuccan	*Cuculus canorus*
Estast	*Estrilda astrild*
Aegcau	*Aegithalos caudatus*
Pormar	*Porphyrio martinicus*
Turhor	*Turdus hortulorum*
Parmaj	*Parus major*
Calalb	*Calidris alba*
Picpic	*Pica pica*
Galgal	*Gallus gallus*
Phacol	*Phasianus colchicus*

*Bird species abbreviation.

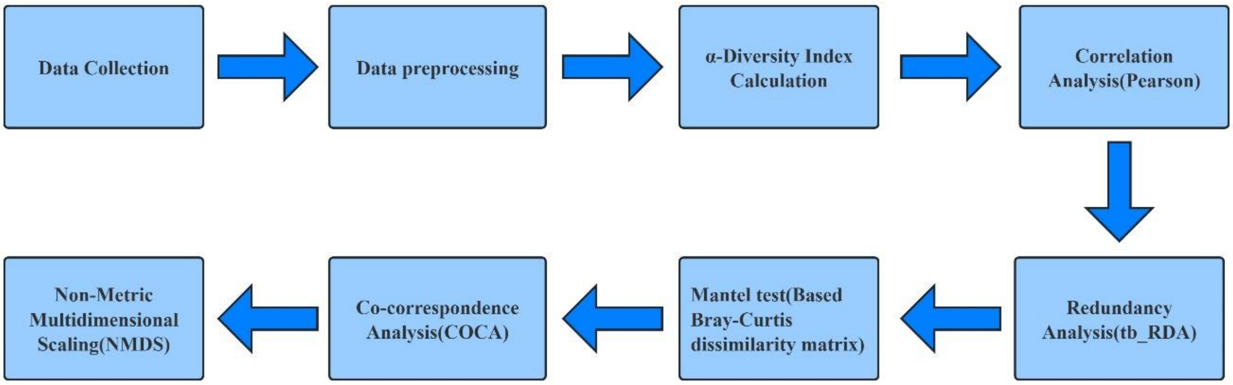

Figure S1. Flowchart of data processing and correlation analysis.

A

B

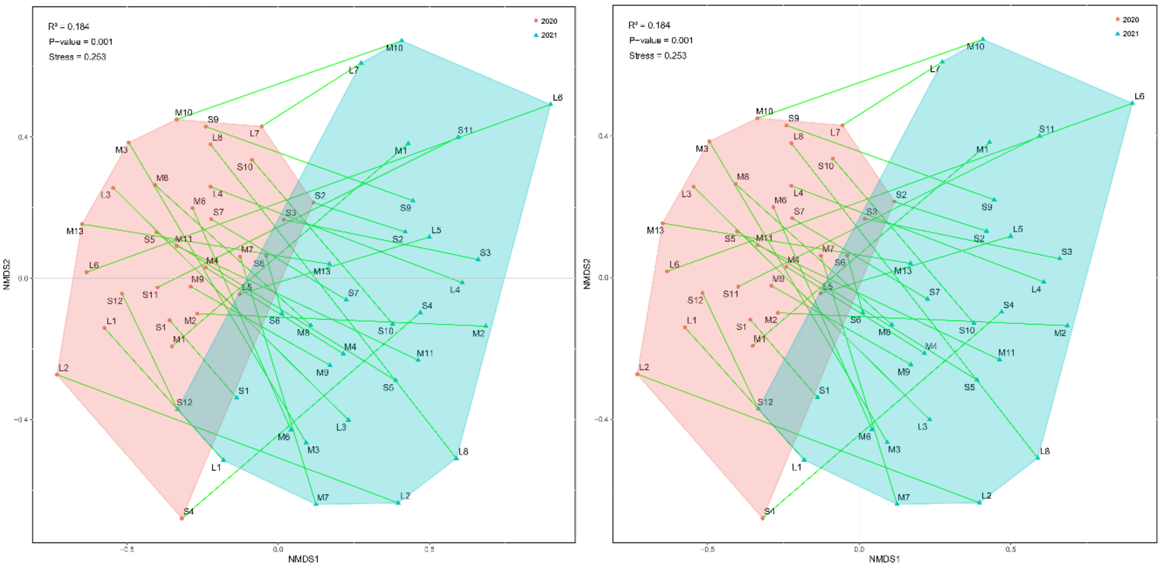

Figure S2. Non-metric multidimensional scaling (NMDS) is based on the Bray–Curtis dissimilarity matrix of different sampling methods for the spider (A) ground pitfall method and (B) suction method. The two sampling years for the same plots are connected by a thin line. Points in the same background color range represent plots sampled in the same year.
